# Plasma and aqueous levels of subfatin, preptin and betatrophin in patients with diabetic retinopathy

**DOI:** 10.1186/s12886-023-03075-0

**Published:** 2023-07-11

**Authors:** Sabiha Güngör Kobat, Fatih Cem Gül, Fatih Çelik, Seda Liman Uzun, Mehmet Ali Kobat, Ramazan Fazıl Akkoç, Süleyman Aydın

**Affiliations:** 1 Department of Ophthalmology, Firat University, Elazig, Turkey; 2 Universal Eye Center, Elazig, Turkey; 3 Department of Ophthalmology, Elazıg Health Science University, Elazıg, Turkey; 4 Department of Cardiology, Firat University, Elazig, Turkey; 5 Department of Anatomy, Medical School, Firat University, Elazig, Turkey; 6 Department of Biochemistry, Firat University, Elazıg, Turkey

**Keywords:** Diabetes mellitus, Retinopathy, Subfatin, Preptin, Betatrophin

## Abstract

**Aim:**

To examine subfatin, preptin and betatrophin levels in plasma and aqueous in patients with diabetes mellitus (DM) (with and without retinopathy).

**Material and method:**

Sixty patients, who were similar in terms of age and gender, and were scheduled for operation due to cataract, were included in the study. The patients were divided into three groups as Group C (20 weeks without diabetes and comorbidity), Group DM (20 patients with DM but no retinopathy) and Group DR (20 patients with diabetic retinopathy). The preoperative body mass index (BMI), fasting plasma glucose, HbA1c, lipid profile levels of all patients in the groups were examined. Blood samples were also taken for plasma subfatin, preptin and betatrophin levels. At the beginning of the cataract surgery, 0.1 ml of aqueous fluid was taken from the anterior chamber. Plasma and aqueous subfatin, preptin and betatrophin levels were analyzed by ELISA (enzyme-linked immunosorbent assays) method.

**Results:**

In our study results, there was a significant difference in BMI, fasting plasma glucose and hemoglobin A1c levels (*p* < 0.05 for all parameters). Plasma and aqueous subfatin levels were higher in Group DR compared to Group C (*p* < 0.001, *p* = 0.036, respectively). Plasma and aqueous preptin levels were higher in group DR and group DM than in group C (*p* = 0.001, *p* = 0.002, *p* < 0.001, *p* = 0.001, respectively). Plasma and aqueous betatrophin levels were higher in Group DR compared to group C (*p* = 0.001, *p* = 0.010, respectively).

**Conclusion:**

Subfatin, preptin and betatrophin molecules may have an important role in the pathogenesis of diabetic retinopathy.

## Introduction

Diabetes mellitus (DM) is a systemic disease which occurs as a result of insulin deficiency or resistance of tissues to insulin and progresses with hyperglycemia. All micro and macrovascular systems may be affected, including the eyes, kidneys, heart and peripheral nerves. Diabetic retinopathy (DR) is the most important preventable and/or treatable cause of blindness observed in the 20–65 years age group and is one of the most important microvascular complications of DM. Microangiopathy is responsible for the physiopathology of DR. Hyperglycemia, type and duration of diabetes, genetic factors, systemic blood pressure, serum lipid levels, obesity and physical activity have been defined as risk factors effective in the development of DR [1, 2]. Chronic hyperglycemia is an important factor in the development and progression of DR. How hyperglycemia leads to DR has not been fully clarified, but it is known that well controlled glucose levels significantly reduce the microvascular complications of DM [3]. In recent years, there has been increasing interest in adipokines originating from adipose tissue because of the effects on glucose and energy metabolism.

Subfatin is an adipokine expressed by adipose tissue and skeletal muscle. It has been shown that subfatin up-regulates anti-inflammatory cytokines in adipose tissue and increases insulin sensitivity in adipose tissue by increasing the expression of peroxisome proliferator-activated receptor gamma (PPAR-γ) and thereby has a role in glucose metabolism [4–6]. The data about the circulation of subfatin levels in diabetic patients are conflicting. Lee et al. [7] reported that subfatin levels are lower in diabetes, while in studies by Chung [8] and Wang [9], higher subfatin levels in diabetic patients were reported.

Preptin is another molecule thought to be effective in glucose metabolism. Preptin has 34 amino acids derived from pro-insulin-like growth factor II (pro-IGF-II), and is a new hormone of peptide structure, which is known to play a role in mineral metabolism [10, 11]. It is expressed from pancreas beta cells together with insulin. An increase or decrease in the concentration of preptin levels in the circulation can correct insulin expression and therefore it is thought to be an amplificator of glucose-mediated insulin expression [12]. Preptin levels are affected by fasting insulin and insulin resistance. Therefore, preptin is thought to be an important factor in the etiology of insulin resistance [13]. Preptin concentration in Type 2 DM patients, obese patients and those with metabolic syndrome has been reported to be higher than that of healthy individuals [14, 15].

Betatrophin is a recently defined adipokine expressed from the liver and adipose tissue, which can affect glucose and lipid metabolism [16]. Plasma betatrophin levels have been reported to be high in DM patients [17]. It is thought that insulin resistance and high insulin levels may contribute to the up-regulation of betatrophin levels. By inhibiting lipoprotein lipase activity, betatrophin may increase plasma triglyceride levels. It is also thought that over-expression of betatrophin could be associated with hypertriglyceridemia [18, 19]. From an extensive literature search, no study could be found that showed the relationship between diabetic retinopathy and subfatin, preptin and betatrophin. These three proteins are involved in the regulation of carbohydrate metabolism. And then again, as remembered disorder of carbohydrate metabolism is generally considered the primary culprit in the development and progression of diabetic retinopathy. So these three proteins are a logical culprit to consider in diabetic retinopathy. Therefore, the aim of this study was to compare plasma and aqueous subfatin, preptin and betatrophin levels in patients with diabetic retinopathy with those of non-diabetics.

## Material and method

The study was conducted in accordance with the principles of the Declaration of Helsinki by obtaining the approval of the Fırat University Faculty of Medicine, Non-invasive Local Ethics Committee (Approval no: 2020/07–10) and informed consent from the participants. The study included patients who presented at the Eye Diseases Polyclinic of Elazig Health Sciences University because of reduced vision, were diagnosed with cataract following a detailed ophthalmological examination, and subsequently underwent cataract surgery. The patients were separated into 3 age and gender-matched groups of 20 cataract patients with no diabetes or additional disease as the control group (C), 20 cataract patients with diabetes and no retinopathy (DM), and 20 cataract patients with diabetic retinopathy (12 proliferative, 8 non-proliferative) (DR). A 10 ml blood was taken from all the patients in the morning after 8–12 h fasting into a tube containing aprotinin (BD Vacutainer SST II Advance, BD, Plymouth, UK) [20].

All the patients were examined with respect to body mass index (BMI: kg/m [2]), fasting plasma glucose (FPG), HbA1c, and lipid profile (LDL, HDL, triglycerides). The patients did not have any macro or microvascular anomalies other than ophthalmologic involvement. The blood samples were centrifuged at 4000 rpm for 10 min and the obtained plasma was placed in small volume tubes and stored at -20 °C until assay for subfatin, preptin and betatrophin. All the patients were applied with Phaco + IOL implantation, and during the cataract operation, aqueous samples were taken and stored at -20 °C until the testing day.

### Surgical method

Phacoemulsification was used throughout this study as described previously [21]. Thirty minutes before the operation, alprazolam (0.5 mg) was orally administered for sedation of the patient. Topical cyclopentolate (1%), tropicamide (0.05%), and phenylephrine (2.5%) were used for pupil dilation. For local anesthesia, topical % 0.5 Propakain HCL dropped on corneal and conjunctival surface. The cornea was incised at the 9 o’clock positions with a 20-G MVR knife and aqueous samples taken from this incision from the anterior chamber. Approximately 0.1 ml of aqueous sample was taken using a 30 Gauge needle. In addition another corneal incision was made at 1 o’clock. Viscoelastic material was inserted. At the 11 o’clock position, a corneal incision was made using a number 2.8 knife. The lens was emulsified with a stable salt solution (BSS), followed by the horizontal chop method with hydrodissection and hydrodelineation. The remaining lens material was removed by manual irrigation and aspiration (I/A) of the cannula. A foldable intraocular lens was installed using a cartridge system. The viscoelastic material inserted into the anterior chamber was removed using the manual I/A method. The incision site was closed with stromal hydration, and any wound leakage was controlled [21].

### Measurement of hormones in biological fluids

Plasma subfatin, preptin, and betatrophin levels were examined using the Human subfatin, preptin, betatrophin ELISA Kit (Sunred Biological Technology, Shanghai, China) in a plate-washing -incubation CombiWash device (Human Diagnostics, Wiesbaden, Germany) in accordance with the study procedures defined in the kit catalogue. Absorbance measurements were taken with a Chromate 4300 Microplate Reader (Awareness Technology, Palm City, USA).

These kit companies in their kit catalogs stated that the minimum detection limit of subfatin was 0.042 ng/mL. The intra-assay and inter-assay coefficients of variation for plasma subfatin were < 10% and < 12%, respectively. The minimum detection limit of preptin was 5.125 ng/L (0.0051 ng/mL). The intra-assay and inter-assay coefficients of variation for plasma preptin were < 10% and < 12%, respectively. The minimum detection limit of betatrophin was 7.334 ng/L (0.0073 ng/mL). The intra-assay and inter-assay coefficients of variation for plasma betatrophin were < 10% and < 15%, respectively. Preptin and betatrophin concentration units are given in ng/L and subfatin in ng/mL by the manufacturer. However, to facilitate the comparison between parameters, all units were converted to ng/mL [20].

### Assay validation of kits for aqueous fluids in our laboratory

Aqueous assay validation was performed according to a previously published method by Aydin [22], as was briefly described below.

### Linearity

Two aqueous liquids and blood samples were diluted (1/2, 1/4, 1/8) with distilled water in order to find the preptin, subfatin and betatrophin linearity.

### Recovery

Two Aqueous liquids and blood samples were enriched with pure amounts of betatrophin, preptin and subfatin. The percentage recovery was calculated as follows: recovered value/expected value × 100.

### The coefficient of variation (CV)

The intra-assay (within-day) and inter-assay variation (between days) were determined for two different two aqueous liquids and blood samples using the means of 2–3 replicates of each. The coefficient of variation (CV) is calculated as: CV = Standard Deviation (SD)/Mean concentration.

### Statistical analysis

Data obtained in the study were analyzed statistically using SPSS version 22.0 software (Statistical Package for the Social Sciences version 22.0, SPSS Inc., Chicago, IL, USA). To determine the significance of the differences between the groups with respect to age, gender, FPG, HbA1c, lipid profile, and plasma and aqueous subfatin, preptin and betatrophin levels, the Mann–Whitney U-test was applied. A value of *p* < 0.05 was accepted as statistically significant.

Since the sample sizes of the Subfatin, Preptin and Betatrofin blood and aqueous C, DM and DR groups were small (*n* < 29), their normality was measured with the Shapiro–Wilk test. In order to compare the triple groups, the difference between the groups were examined by using the Kruskal–Wallis test, since all three groups were not normal at the same time. In order to determine which group(s) caused this difference when there was a difference between the groups in the Kruskal–Wallis test, since the two groups to be compared were not all normal at the same time, the Mann–Whitney U-test was used.

## Results

The validation of the kits, we use has been made in our laboratory. Results of the linearity of used kits in biological samples were summarized in Table 1. Table 2 indicated recovery assay results of Kits used through this study. Also, in our laboratory intra assay values were calculated < %10 for subfatin, preptin and betatrophin, inter assay values were recorded as < 15% for subfatin, preptin, and betatrophin, respectively.

**Table 1:** Linearity of Kits in biological samples used through this study

		**Undiluted (100%)**	**1/2**	**1/4**	**1/8**
**Subfatin (ng/mL)**	Aqueous-1	125.8	122.4(97.2%)	118.2 (93.9%)	136.2 (108%)
	Aqueous-2	117.9	124.2 (105%)	112.2 (95.1%)	116.6(98.8%)
	Blood-1	98.4	94.8 (96%)	104.4 (106%)	102.8 (104%)
	Blood-2	94.5	106.2 (112%)	92.8 (98.2%)	102.2 (108%)
**Preptin (ng/L)**	Aqueous-1	365.4	388.4 (105%)	366.4 (100%)	372.4 (102%)
	Aqueous-2	452.7	442.4(97.7%)	472.8 (104%)	466.4 (103%)
	Blood-1	266.4	272.8 (102%)	278.6 (105%)	294.6 (111%)
	Blood-2	380.0	366.8 (96.5%)	388.6 (102%)	392.2 (103%)
**Betatrophin (ng/L)**	Aqueous-1	2.45	2.66 (108%)	2.32(94.6%)	2.26 (92.2%)
	Aqueous-2	2.74	2.82 (102%)	2.88 (105%)	2.68 (97.8%)
	Blood-1	2.14	2.16 (100%)	2.02 (94.3%)	2.12 (100%)
	Blood-2	2.18	2.12 (97.2%)	1.98 (90.8%)	2.28 (104%)

**Table 2:** Recovery Assay of used kits in this study

		**Initial concentration**	**Added**	**Recovered**	**Expected**	**Recovery** **(%)**
**Subfatin (ng/mL)**	Aqueous-1	109.51	300	414.4	409.51	108
	Aqueous-2	137.15	300	443.6	437.15	101
	Blood-1	91.20	300	389.8	391.20	102
	Blood-2	85.60	300	424.6	385.60	90
**Preptin (ng/L)**	Aqueous-1	266.49	300	582.4	566.49	115
	Aqueous-2	205.85	300	516.8	505.85	102
	Blood-1	175.53	300	486.2	475.53	102
	Blood-2	212.23	300	528.6	512.23	103
**Betatrophin (ng/L)**	Aqueous-1	2.55	8	10.98	10.55	104
	Aqueous-2	2.88	8	11.02	10.88	102
	Blood-1	2.13	8	10.96	10.13	108
	Blood-2	2.06	8	11.02	10.06	109

The 20 cataract patients with no diabetes or additional disease (C) comprised 11 females (55%) and 9 males (45%) with a mean age of 66.55 ± 10.30 years. The group of 20 cataract patients with diabetes and no retinopathy (DM) comprised 12 females (60%) and 8 males (40%) with a mean age of 67.10 ± 9.01 years. The group of 20 cataract patients with diabetic retinopathy (DR) comprised 12 females (60%) and 8 males (40%) with a mean age of 65.10 ± 8.34 years. No statistically significant difference was determined between the groups with respect to age and gender (*p* > 0.05 for all) (Table 3).

**Table 3:** Demographic characteristics of the patients in all the groups

	**C**	**DM**	**DR**
Age (years)	66.55 ± 10.30	67.10 ± 9.01	65.10 ± 8.34
Gender (F/M)	11F/9 M	12F/8 M	12F/8 M
BMI (kg/m^2^)	26.10 ± 1.41	29.40 ± 2.94^a^	31.45 ± 2.94^b^
FPG (mg/dL)	95.05 ± 9.82	162.70 ± 22.87^a^	229.25 ± 44.06^b^
HbA1c (%)	5.30 ± 0.43	7.13 ± 0.37^a^	8.25 ± 1.00^b^
HDL (mg/dL)	47.50 ± 8.12	41.85 ± 5.94^a^	40.80 ± 7.45^a^
LDL (mg/dL)	112.65 ± 17.95	138.10 ± 17.81^a^	146.15 ± 15.12^a^
Triglyceride (mg/dL)	138.10 ± 23.34	192.10 ± 54.78^a^	195.10 ± 53.51^a^

*C* Cataract, *DM* Diabetes mellitus, *DR* Diabetic retinopathy, *BMI* Body mass index, *FPG* Fasting plasma glucose, *HbA1c* Hemoglobina1c, *HDL* High density lipoprotein, *LDL* Low density lipoprotein, *F* Female, *M*, Male

^a^Compared with Group C *p* < 0.05 (Mann Whitney U-test)

^b^Compared with Group C and Group DM *p* < 0.05 (Mann Whitney U-test)

The fasting plasma glucose levels of groups C, DM, and DR were determined as 95.05 ± 9.82 mg/dL, 162.70 ± 22.87 mg/dL, 229.25 ± 44.06 mg/dL, respectively. The HbA1c levels of groups C, DM, and DR were determined as 5.30 ± 0.43, 7.13 ± 0.37, 8.25 ± 1.00, respectively. The glucose and HbA1c levels of group DR were determined to be statistically significantly higher than those of group C and group DM (*p* < 0.001, *p* < 0.001). The BMI values of groups C, DM, and DR were determined as 26.10 ± 1.41, 29.40 ± 2.94, 31.45 ± 2.94, respectively. The BMI values of group DR were determined to be statistically significantly higher than those of group C and group DM (*p* < 0.05, *p* < 0.05) (Table 3).

The HDL levels in groups C, DM and DR were determined as 47.50 ± 8.12 mg/dL, 41.85 ± 5.94 mg/dL, and 40.80 ± 7.45 mg/dL, respectively. The LDL levels in groups C, DM and DR were determined as 112.65 ± 17.95 mg/dL, 138.10 ± 17.81 mg/dL L, and 146.15 ± 15.12 mg/dL, respectively. Triglyceride levels were determined in groups C, DM and DR as 138.10 ± 23.34 mg/dL, 192.10 ± 54.78 mg/dL, and 195.10 ± 53.51 mg/dL, respectively. The LDL and triglyceride levels in group DM and group DR were determined to be statistically significantly higher than those of group C (*p* < 0.05, *p* < 0.05). There was no statistically significant difference between group DM and group DR (*p* > 0.05). The HDL levels in group DM and group DR were determined to be statistically significantly lower than those of group C (*p* < 0.05, *p* < 0.05). There was no statistically significant difference between group DM and group DR (*p* > 0.05) **(**Table 3**)**.

**Fig. 1:**
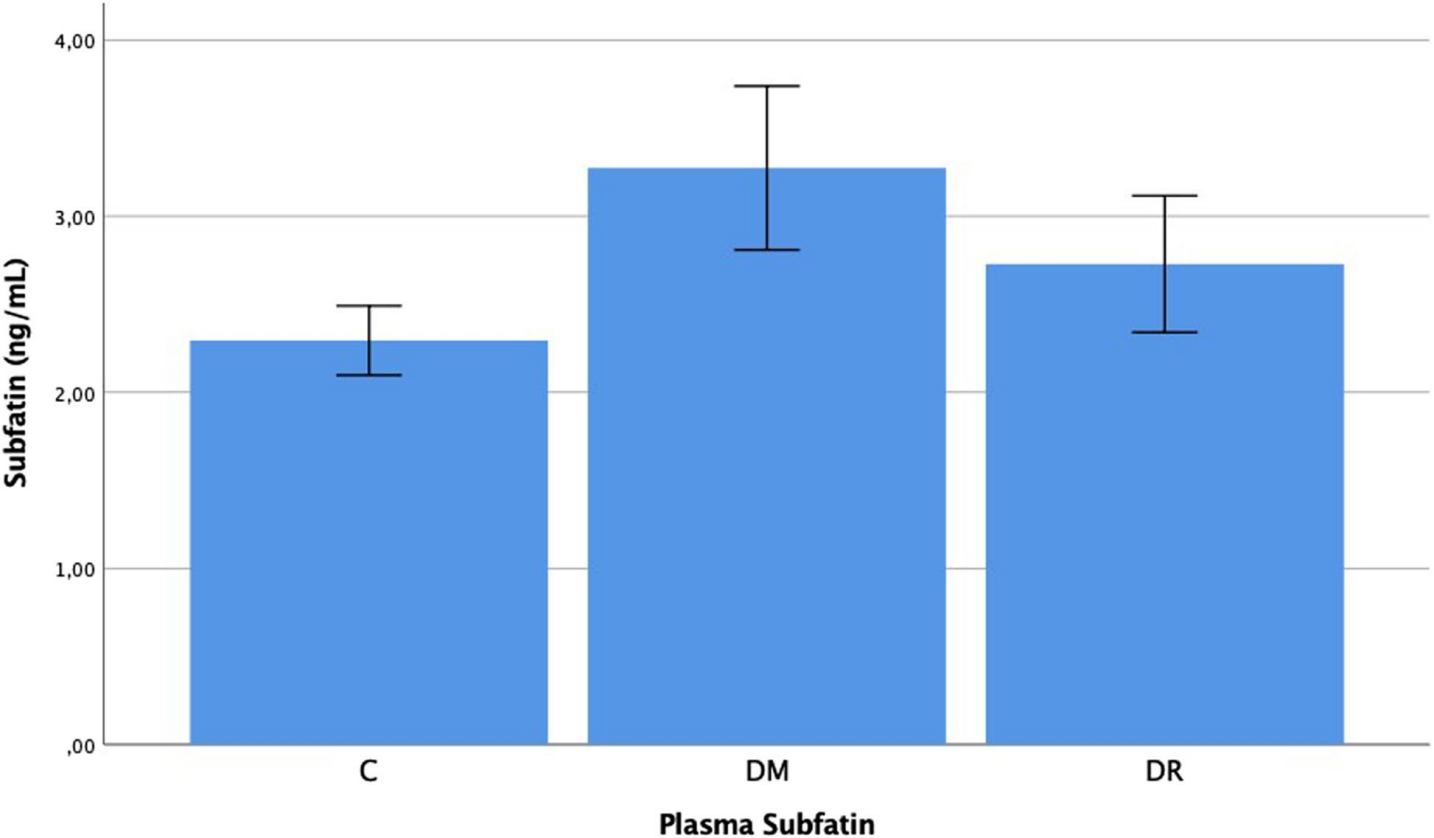
Subfatin levels in the plasma of patient with C, DM, DR, C: Cataract; DM: Diabetes mellitus; DR: Diabetic retinopathy. -Mann Whitney U-test

**Fig. 2:**
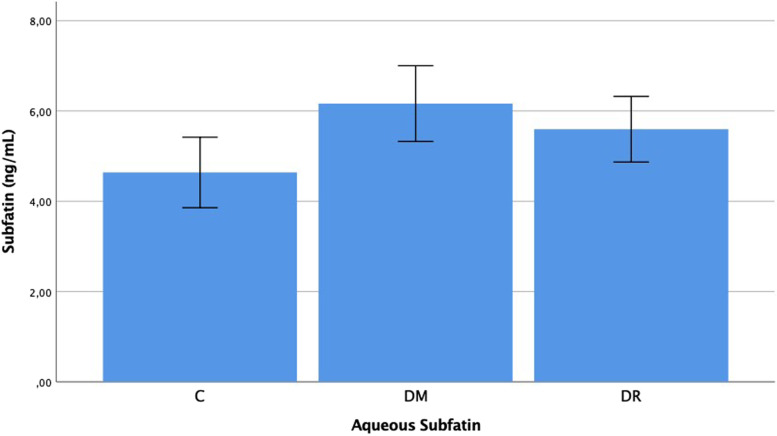
Subfatin levels in the aqueous of patient with C, DM, DR, C: Cataract; DM: Diabetes mellitus; DR: Diabetic retinopathy. -Mann Whitney U-test

The plasma subfatin levels were determined as 2.293 ± 0.420 ng/mL in group C, 2.728 ± 0.829 ng/mL in group DM and 3.273 ± 0.994 ng/mL in group DR. The aqueous subfatin levels were determined as 4.638 ± 1.669 ng/mL in group C, 5.595 ± 1.553 ng/mL in group DM, and 6.164 ± 1.792 ng/mL in group DR. The plasma and aqueous subfatin levels in group DR were determined to be statistically significantly higher than those of group C (*p* < 0.001, *p* = 0.036). No statistically significant difference was determined between group DM and group C with respect to the plasma and aqueous subfatin levels (*p* = 0.130, *p* = 0.093). In the comparison between group DM and group DR, a significant difference was observed in the plasma levels and no significant difference was determined in the aqueous levels (*p* = 0.031, *p* = 0.203) (Figs. 1 and 2).

**Fig. 3:**
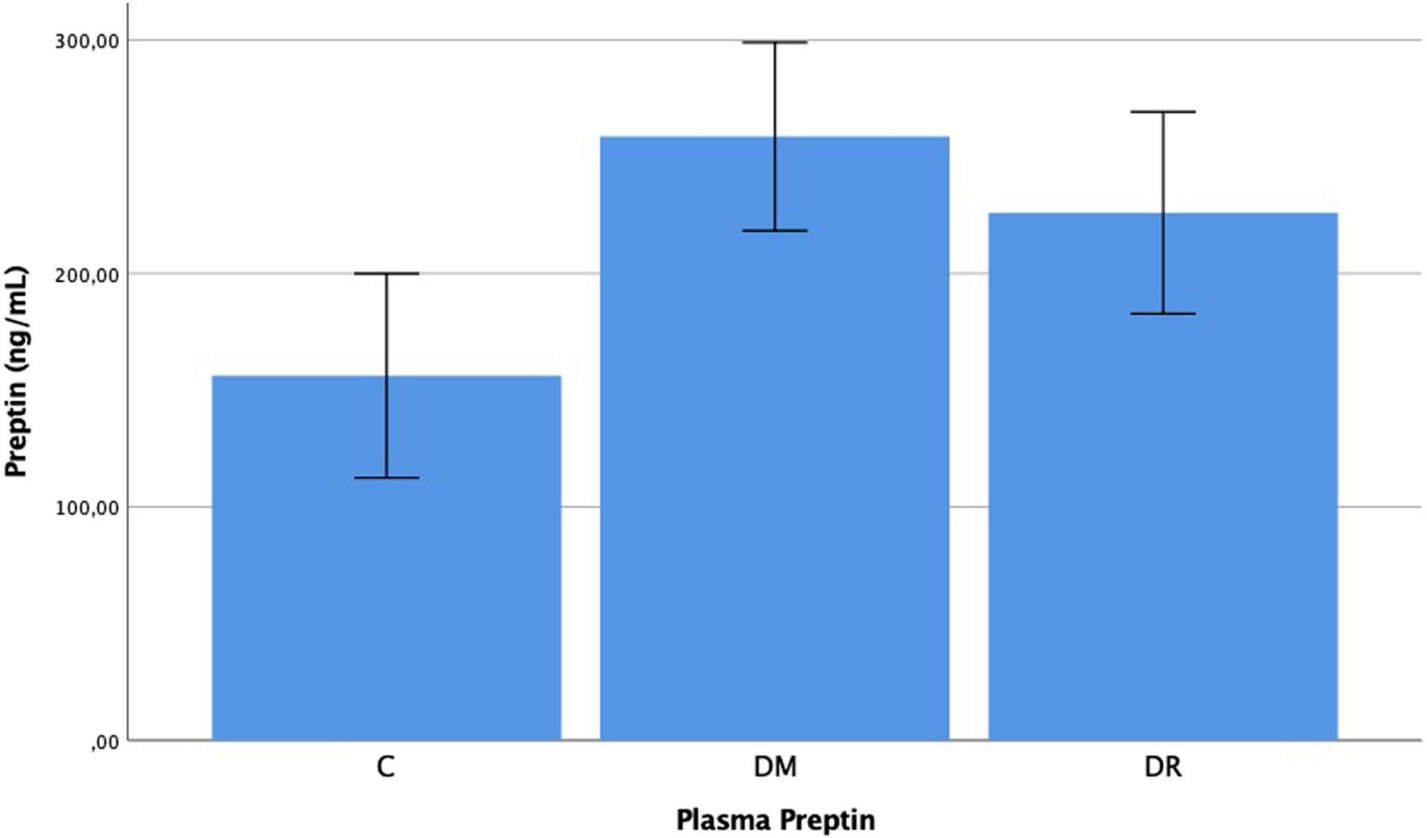
Preptin levels in the plasma of patient with C, DM, DR, C: Cataract; DM: Diabetes mellitus; DR: Diabetic retinopathy. -Mann Whitney U-test

**Fig. 4:**
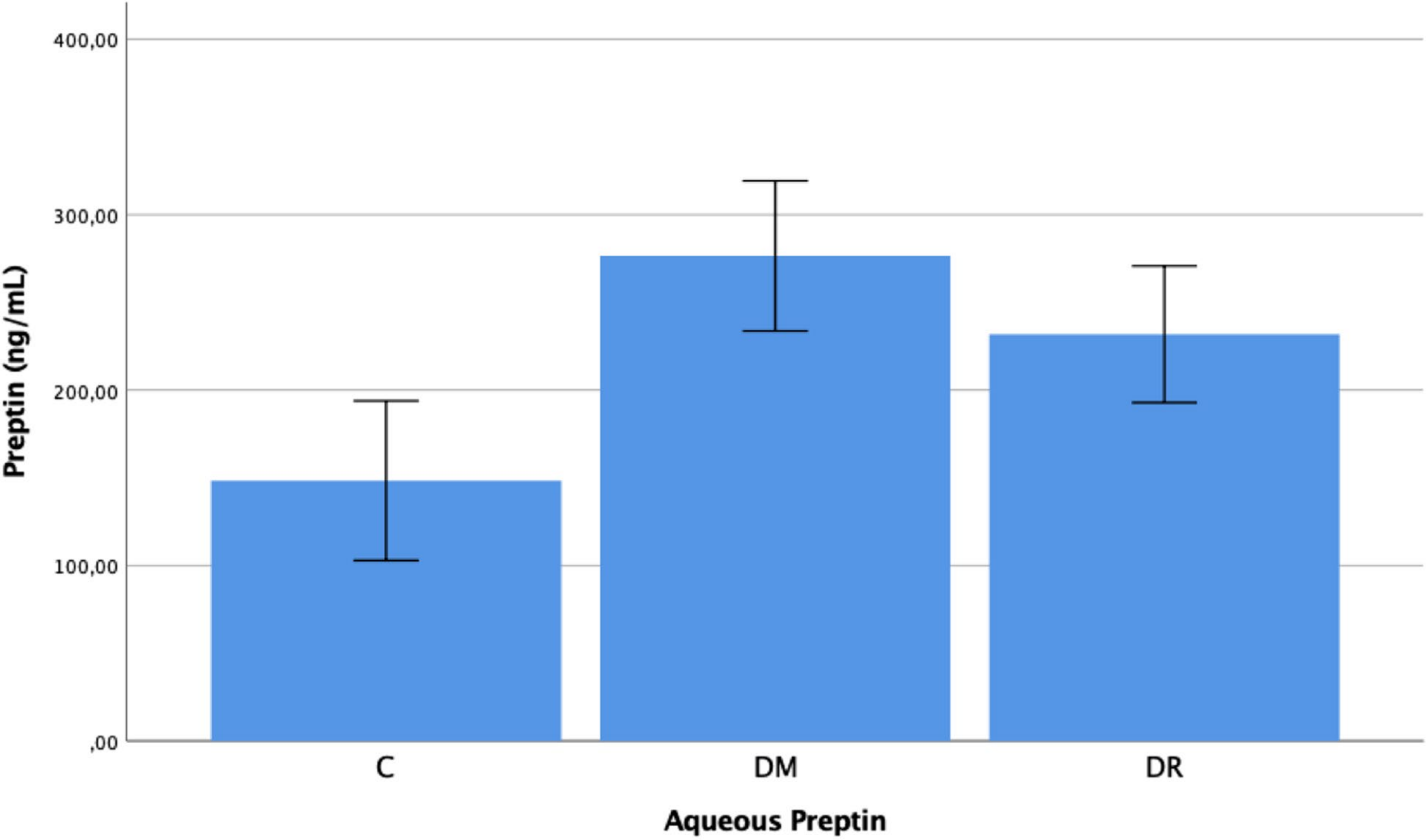
Preptin levels in the aqueous of patient with C, DM, DR, C: Cataract; DM: Diabetes mellitus; DR: Diabetic retinopathy. -Mann Whitney U-test

The plasma preptin levels were determined as 0.157 ± 0.094 ng/mL in group C, 0.226 ± 0.093 ng/mL in group DM, and 0.259 ± 0.086 ng/mL in group DR. The aqueous preptin levels were determined as 0.1484 ± 0.097 ng/mL in group C, 0.232 ± 0.083 ng/mL in group DM, and 0.277 ± 0.091 ng/mL in group DR. The plasma and aqueous preptin levels in group DR and group DM were determined to be statistically significantly higher than those of group C (*p* = 0.001, *p* = 0.002, *p* < 0.001, *p* = 0.001, respectively). No statistically significant difference was determined between group DR and group DM with respect to plasma and aqueous preptin levels (*p* = 0.293, *p* = 0.107) (Figs. 3 and 4).

**Fig. 5:**
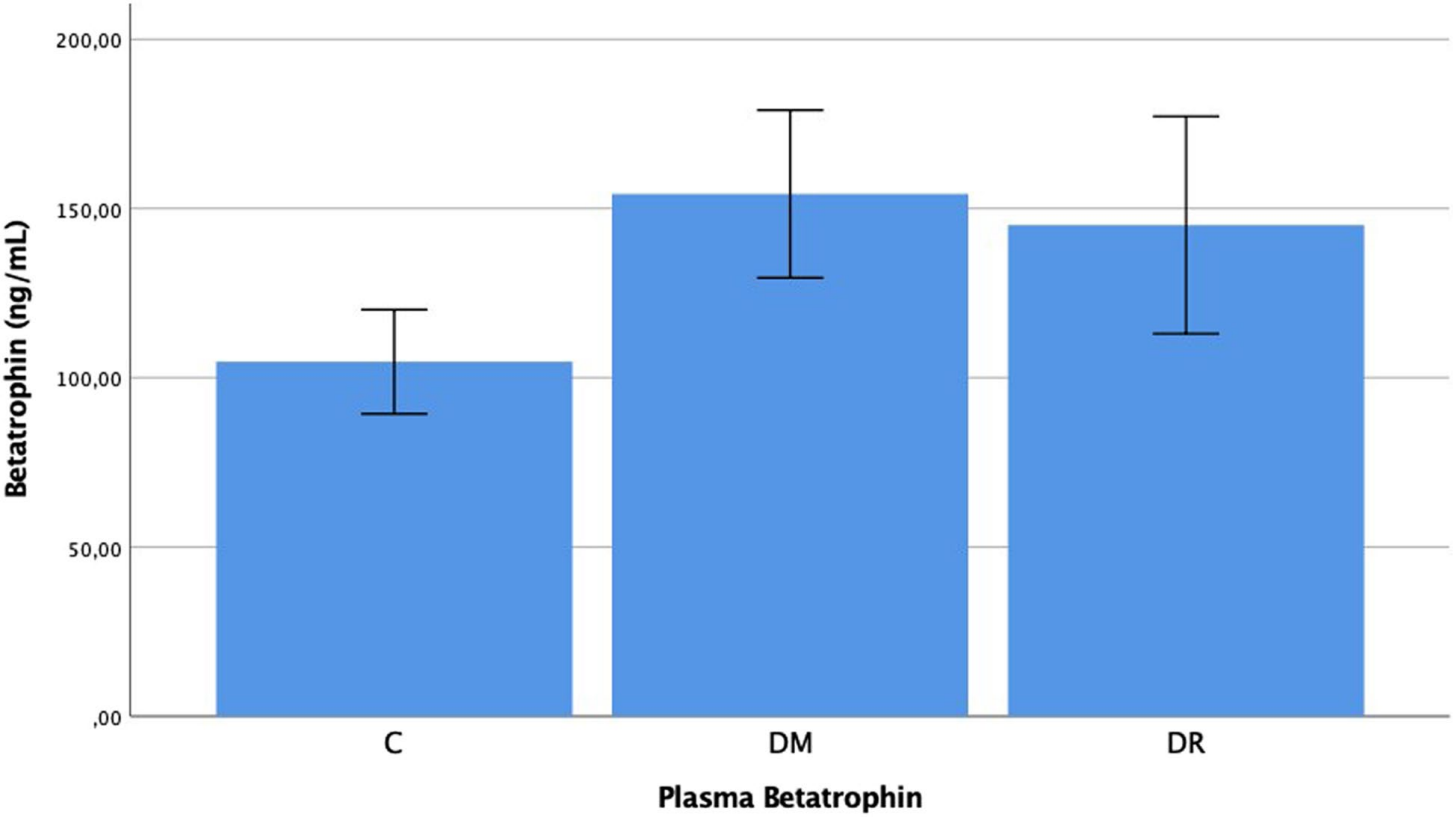
Betatrophin levels in the plasma of patient with C, DM, DR, C: Cataract; DM: Diabetes mellitus; DR: Diabetic retinopathy. -Mann Whitney U-test

**Fig. 6:**
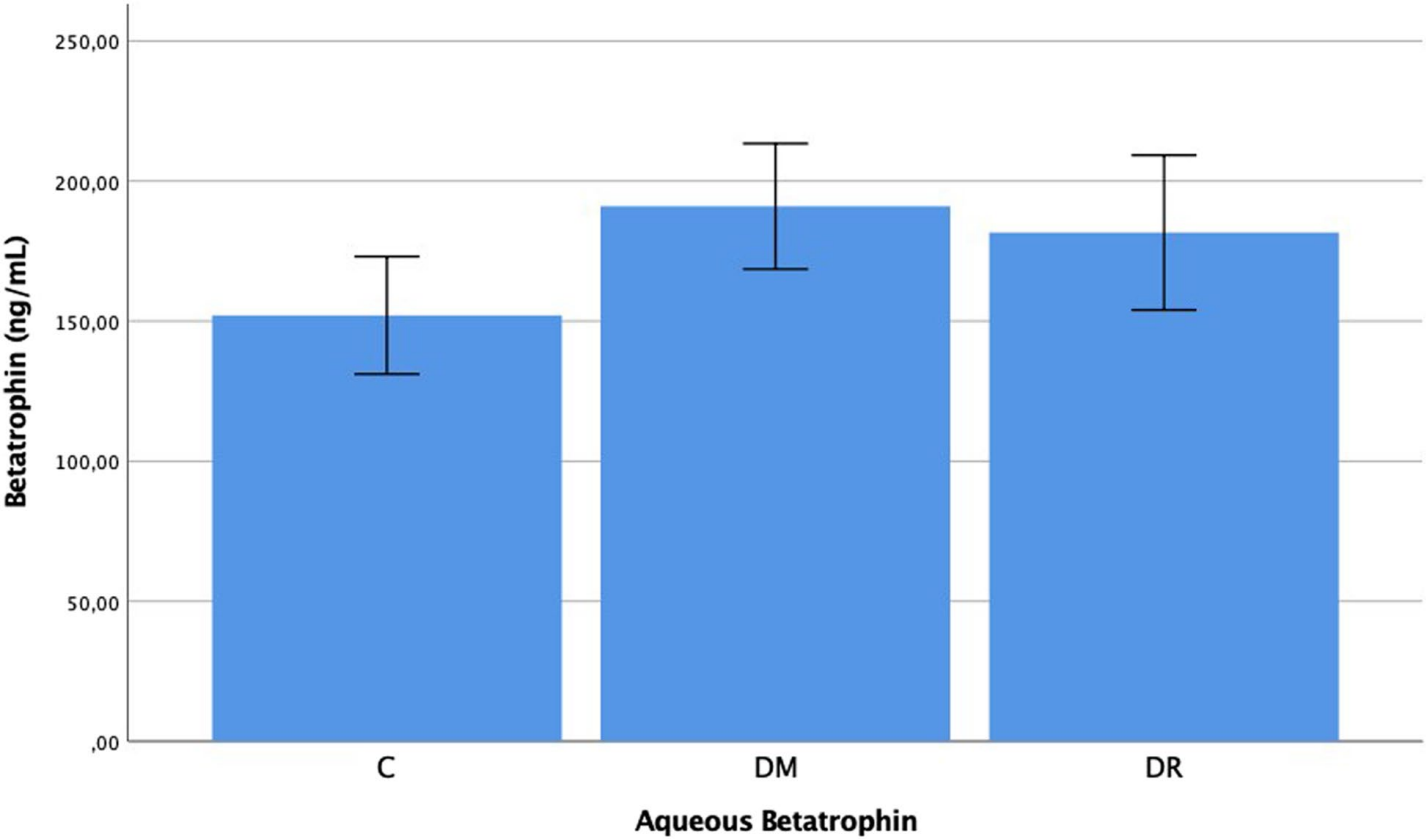
Betatrophin levels in the aqueous of patient with C, DM, DR, C: Cataract; DM: Diabetes mellitus; DR: Diabetic retinopathy. -Mann Whitney U-test

The plasma betatrophin levels were determined as 0.105 ± 0.033 ng/mL in group C, 0.145 ± 0.069 ng/mL in group DM, and 0.154 ± 0.053 ng/mL in group DR. The aqueous betatrophin levels were determined as 0.152 ± 0.045 ng/mL in group C, 0.182 ± 0.059 ng/mL in group DM, and 0.191 ± 0.048 ng/mL in group DR. The plasma and aqueous betatrophin levels in group DR were determined to be statistically significantly higher than those of group C (*p* = 0.001, *p* = 0.010). In the comparison between group DM and group C, a significant difference was observed in the plasma levels and no significant difference was determined in the aqueous levels (*p* = 0.025, *p* = 0.068). No statistically significant difference was determined between group DR and group DM with respect to plasma and aqueous betatrophin levels (*p* = 0.194, *p* = 0.239) (Figs. 5 and 6).

## Discussion

The results of this study showed that the fasting plasma glucose levels, HbA1c levels and BMI values were significantly higher in group DR than in group DM and group C. Hyperglycemia is a risk factor which can be changed and is important in the development of DR. Free radicals which form as a result of oxidative stress and advanced glucose metabolism products resulting from a lengthy hyperglycemic phase, lead to changes in the capillary. Endothelial damage of the capillaries, thickening of the basal membrane and increased thrombocyte aggregation lead to retinal ischemia secondary to vaso-occlusion. When retinal ischemia continues, DR enters the proliferative phase with the development of secondary neovascular tissues. Preretinal proliferative fibrotic membranes developing as a result of fibroblastic activity progress to tractional retinal detachment together with neovascular membranes, and the burn-out stage, which is the final stage, and consequently, light sensitivity is lost. Despite tight blood glucose regulation and developed medical (intravitreal drugs) and surgical (laser photocoagulation and vitreoretinal surgery) techniques, these do not prevent the sight loss of DR patients at the desired rates [23, 24]. Therefore, studies of the etiology and treatment are ongoing. With this purpose, the current study was planned to examine the plasma and aqueous levels of subfatin, preptin and betatrophin.

When the subfatin results of this study are examined, the plasma subfatin levels of the DR group were determined to be statistically significantly higher than those of group DM and group C. In addition, the aqueous subfatin levels of group DR were significantly higher than those of the control group, group C (*p* < 0.05). Previous studies conducted on the subfatin levels in diabetes have provided conflicting data. In a study by Lee et al. [7] subfatin levels were reported to be lower in diabetes. As in the current study, Chung [8] and Wang [9] reported that subfatin levels were higher in diabetes.

Subfatin is an adipokine expressed by adipose tissue and skeletal muscle. By increasing PPAR-γ expression, subfatin increases insulin sensitivity in adipose tissue and is therefore thought to be effective in glucose metabolism [4, 5]. However, this does not explain the high subfatin levels in diabetes. It has been suggested that over-expression of subfatin may cause hyperinsulinemia and insulin resistance as a result of a reduction in lipogenesis and inhibition of PPAR-γ expression in adipocytes [6]. The developing insulin resistance causes the tissues to be exposed to high glucose and may play a role in the pathogenesis of DR development. The high subfatin levels determined in this study may have played a role in DR pathogenesis through this mechanism.

When the preptin results of the study are examined, the plasma and aqueous preptin levels of groups DR and DM were observed to be higher than those of group C (*p* < 0.05). Previous studies have shown that preptin concentration in the circulation is higher in Type 2 DM patients, obese patients and those with metabolic syndrome [13–15]. Preptin is a new hormone of peptide structure derived from pro-IGF-II, which is expressed from pancreas beta cells together with insulin. It has been shown that preptin is released from rat pancreas beta cells together with insulin in response to an increased glucose level. It has been thought that insulin expression can be corrected by an increase or decrease in the concentration of circulation of preptin levels [10–12]. In addition, preptin levels are affected by fasting insulin and insulin resistance [13]. In rats, it has been determined that intravenous preptin infusion during glucose intake together with insulin leads to reduced glucose levels. From the results of that study, it was reported that through the protein kinase c and phospholipase c pathways of IGF-2R activated with preptin, insulin expression was increased as a response to increased glucose levels [25]. In the current study, the preptin levels were found to be higher in diabetic groups. This could be associated with the expression of preptin together with insulin from the pancreas, secondary to hyperglycemia. It is known that well controlled glucose levels can significantly reduce the progression of DR. In the future, preptin treatment could be considered an alternative in the treatment of DR.

Finally, when the betatrophin results of the current study are examined, the plasma and aqueous betatrophin levels of the DR group were observed to be higher than those of the control group, group C (*p* < 0.05). Previous studies have shown that blood betatrophin levels were high in patients with DM, gestational DM and polycystic ovary syndrome (PCOS) [16, 17, 26–31]. Betatrophin is an adipokine expressed from the liver and adipose tissue and is thought to increase pancreas beta cell proliferation and insulin resistance. It is also thought to inhibit lipoprotein lipase activity and increase triglyceride levels [16–19]. It has been shown to be an important risk factor in the development of DR [32, 33]. Consistent with previous findings in the literature, LDL cholesterol and triglyceride levels in the current study were observed to be high in diabetic patients. Betatrophin may play a significant role in the pathogenesis of DR as it both increased insulin resistance and caused a hyperlipidemia table. In conclusion, there is some correlation between levels of subfatin, preptin and betatrophin and the existence of diabetic retinopathy in the patient. Therefore, these proteins may play a role in the pathogenesis of diabetic retinopathy. Nevertheless, there is a need for further comprehensive studies with larger groups to be able to determine the role of these molecules in the pathogenesis of diabetic retinopathy and to evaluate the contribution to treatment.

## Data Availability

All data are included in this paper.
